# A Switchable Gold Catalyst by Encapsulation in a Self‐Assembled Cage

**DOI:** 10.1002/chem.201603162

**Published:** 2016-09-04

**Authors:** Anne C. H. Jans, Adrián Gómez‐Suárez, Steven P. Nolan, Joost N. H. Reek

**Affiliations:** ^1^ Homogeneous, Bioinspired and Supramolecular Catalysisvan ‘t Hoff Institute for Molecular SciencesUniversity of AmsterdamScience Park 9041098 XHAmsterdamthe Netherlands; ^2^ School of ChemistryUniversity of St AndrewsSt AndrewsKY16 9STUK; ^3^ Department of Inorganic and Physical ChemistryGhent UniversityKrijgslaan 281 - S39000GentBelgium; ^4^ Chemistry DepartmentCollege of ScienceKing Saud university PO Box 2455Riyadh11451Saudi Arabia

**Keywords:** catalyst encapsulation, catalytic switch, dual catalysis, gold catalysis, supramolecular chemistry

## Abstract

Dinuclear gold complexes have the ability to interact with one or more substrates in a dual‐activation mode, leading to different reactivity and selectivity than their mononuclear relatives. In this contribution, this difference was used to control the catalytic properties of a gold‐based catalytic system by site‐isolation of mononuclear gold complexes by selective encapsulation. The typical dual‐activation mode is prohibited by this catalyst encapsulation, leading to typical behavior as a result of mononuclear activation. This strategy can be used as a switch (on/off) for a catalytic reaction and also permits reversible control over the product distribution during the course of a reaction.

There is a growing interest in transition‐metal catalysis in confined spaces as the approach provides an additional tool to control selectivity and activity in catalysis.^1^ For example, it has been demonstrated that the encapsulation of rhodium complexes in a hemispherical porphyrin assembly results in catalysts with increased activity and unprecedented branched selectivity in the hydroformylation of terminal and internal alkenes.^2^ In addition, the encapsulation of transition‐metal complexes in preformed cavities through weak interactions can lead to unexpected reactivity: M_4_L_6_ anionic tetrahedral capsules, for instance, can host cationic organometallic catalysts in their hydrophobic cavities,^3^ thereby inducing substrate selectivity.^4^ Moreover, reaction rates^5^ and product distribution^6^ can be greatly affected by catalyst encapsulation. Interestingly, many catalytic reactions operate through a dinuclear mechanism^7^ or deactivate via a dinuclear pathway;^8^, ^9^ by encapsulation of a transition metal complex, such decomposition pathways can be suppressed, leading to higher catalytic turnover numbers.^10^

In principle, an encapsulation event could also change the catalytic pathway of a reaction, and as such can be used as a switch for a catalytic transformation. Switchable catalysis is an interesting upcoming field of research as it provides new tools to control the reaction process with external stimuli such as light, pH, or metal coordination, a concept that is important to control reactions in nature.^11^ In a recent study, encapsulation of a photoredox catalyst was shown to be a feasible stimulus to steer reactivity.^12^ In a previous paper we reported the encapsulation of an N‐heterocyclic carbene (NHC) mononuclear gold(I) complex inside a self‐assembled hexameric resorcin[4]arene cage **1**_6_ and showed that the encapsulated catalyst gives a different product distribution than the free complex.^13^ This supramolecular complex is formed in apolar, water‐saturated solvents (Scheme 1).^14^, ^15^ In the current contribution, we report how we change the active gold complex from dinuclear to mononuclear by reversible encapsulation and demonstrate that this can be used for both switching on/off a reaction, as well as for controlling its selectivity during the course of a reaction. Changing the reactivity or selectivity of a gold catalyst has been shown before by changing the ligands^16^ or Brønsted acid/base effects^17^ and even by guest binding by a rotaxane,^18^ but reversibly changing the active species of a gold‐catalyzed reaction as a result of encapsulation has, to the best of our knowledge, not been shown before.

**Scheme 1:**
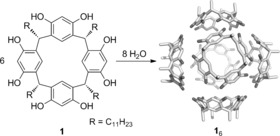
Formation of the self‐assembled hexameric resorcin[4]arene cage.

In this study we use a dinuclear hydroxyl‐bridged gold complex [{Au(NHC)}_2_(μ‐OH)][X] instead of a mononuclear gold complex.^19^ This complex reacts through different mechanisms than mononuclear gold complexes^20^ and is a privileged catalyst for dual activation reactions.^21^ It, however, is too large to fit inside the cage **1**_6,_ 22 and should be split into mononuclear complexes upon encapsulation. The NHC‐Au‐X fragment displays the traditional Lewis acidic character of mononuclear cationic gold catalysts, activating unsaturated substrates such as alkynes through π‐coordination, making them more electrophilic and susceptible towards nucleophilic attack.^23^ Meanwhile, the NHC‐Au‐OH species behaves as a Brønsted base and is known to σ‐activate substrates such as terminal alkynes or phenols.^24^ Together the two species, NHC‐Au‐X and NHC‐Au‐OH, can dually activate substrates through both π‐ and σ‐activation (Scheme 2). This dual‐activation mode was originally proposed by Toste and Houk^25^ and is well established and employed nowadays. In addition, this dual activation has elegantly been explored by Hashmi^26^ and Zhang^27^ for the formation of gold acetylides, which can react as a nucleophile and attack the π‐activated bond of the substrate, and as such represents a common strategy in gold‐mediated synthesis.

**Scheme 2:**
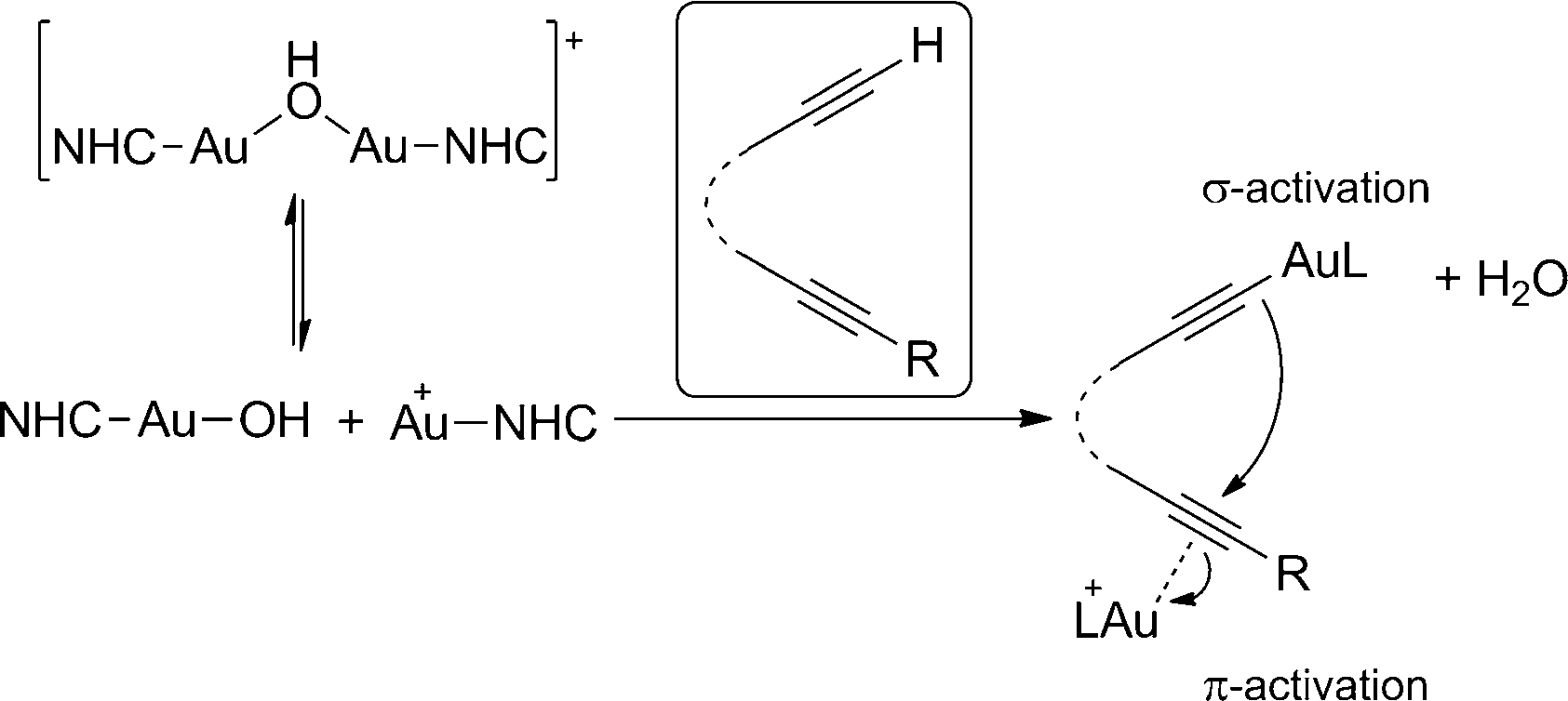
Dinuclear complexes [{Au(NHC)}_2_(μ‐OH)][X] can dually activate substrates.

We envisioned that encapsulation of the two fragments of the dinuclear gold catalyst [{Au(NHC)}_2_(μ‐OH)][X] in separate cages would alter its typical reactivity as they undergo transformations in a site isolated fashion. We anticipated that by complex encapsulation we could reversibly switch between the dinuclear and mononuclear catalyst and, therefore, have a tool to shift from dual gold catalysis to a mononuclear reaction mechanism. This forms the basis for an on/off switchable system, but can also alter the product distribution during a gold‐catalyzed transformation.

Indeed, when complex [{Au(IPr)}_2_(μ‐OH)][BF_4_] **2** (Scheme 3) and the capsule were mixed together, encapsulation of a mononuclear gold complex was confirmed by ^1^H NMR and ^1^H 2D DOSY NMR (see the Supporting Information). To demonstrate the principle of switching catalyst activity by selective encapsulation, the dual gold‐catalyzed hydrophenoxylation reaction was studied.^21^, ^28^ This reaction requires σ‐activation of a phenol by the NHC‐Au‐OH moiety and π‐activation of an alkyne by the cationic NHC‐Au‐X fragment (Scheme 4). Indeed, it was observed that the standard reaction between phenol (**4**) and diphenylacetylene (**5**) readily takes place when using **2** as catalyst in the absence of the cage (full conversion within 60 min). However, in the presence of the cage, the dinuclear complex **2** is broken and encapsulated as mononuclear species. As the dual reaction pathway is no longer available, no conversion to vinyl ether **6** is obtained, even after 24 h.^29^ We wondered if the reaction could be switched on again by adding a competing guest that would bind more strongly to the cage than the gold catalyst. For this purpose we selected tetraalkylammonium salts, as they are known to bind very strongly inside the hexamer.^15^ Gratifyingly, upon addition of Et_4_N^+^BF_4_^−^ (**7**) to expel the gold catalyst from the cage, the catalytic activity was restored and product **6** was obtained in 89 % yield after one hour.

**Scheme 3:**
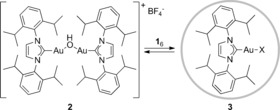
Encapsulation of [{Au(IPr)}_2_(μ‐OH)][BF_4_] in capsule **1**_6_.

**Scheme 4:**
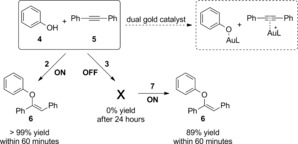
Switching the gold‐catalyzed hydrophenoxylation off and on. Reaction conditions: solvent=toluene, *T*=80 °C, [**4**]=550 mm, [**5**]=500 mm, [**2**]=2.5 mm (0.025 mol %), [**1**]=50 mm.

Next we explored the cage effect on changing the product distribution of a reaction. For this purpose we explored the conversion of 4‐phenyl‐1‐butyne (**8**). Depending on the available reaction pathways, the transformation can yield up to four products (Table 1). In the absence of the cage, **2** can activate two substrates in a σ‐ and π‐activation mode and, therefore, dimerization of **8** takes place through a dual‐activation mechanism, yielding the branched and linear products, **11** and **12** respectively (Table 1, entry 1; Scheme 5).26b, 30 As anticipated, this dual‐activation reaction pathway is completely blocked upon encapsulation of the catalyst and the production of **11** and **12** stopped. Instead, when in the cage, the catalyst produced the intramolecularly cyclized product **10**, which is known to form via a mononuclear pathway inside the cage (Table 1, entry 2).^13^ In the presence of the cage and the competing guest **7**, product **10** was not formed and dimerization products **11** and **12** were obtained (Table 1, entry 3), indicating that under these conditions the catalysis proceeds via a dual‐gold mechanism and thus takes place outside the cage.

**Table 1:** Gold catalyzed conversion of 4‐phenyl‐1‐butyne.^[a]^

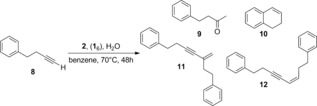						
Entry	**1**_6_	**7**	Yield **9** [%]	Yield **10** [%]	Yield **11** [%]	Yield **12** [%]
1	−	−	7	0	38	8
2	+	−	3	16	0	0
3	+	+	47	0	28	4
4	−	+	6	0	47	8

[a] Reaction conditions: [**8**]=66 mm, [**2**]=1.7 mm (2.5 mol %), [H_2_O]=44 mm, [**1**]=33 mm, [**7**]=33 mm. Yields are averaged from two measurements.

**Scheme 5:**
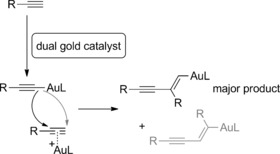
Dimerization of terminal alkynes through σ‐ and π‐activation.

Due to the presence of water molecules to facilitate the self‐assembly of the capsule, the well‐documented hydration product **9** was formed in all cases through π‐activation of the triple bond,^31^, ^32^ but only in minor amounts. We observed an increased rate of formation of this product in the presence of both cage and competing guest **7**, which is interesting but difficult to explain. The addition of only the competing guest **7**, in the absence of cage but with the same amount of water present, does not lead to increased formation of product **9** (Table 1, entry 4).

Next we investigated whether it would be possible to switch the product selectivity during the course of the reaction (Figure 1). The reaction was initiated using only the dinuclear gold catalyst **2**. Under these reaction conditions, the branched dimer **11** was the main product formed, indicating that the dual activation pathway is dominating. After 3 h, an excess of cage **1_6_** was added, thereby removing the dinuclear gold complex from the reaction mixture by encapsulation of the cationic mononuclear gold fragment. The formation of **11** immediately stopped, while production of compound **10** started. After another 3 h, compound **7** was added as a competing guest for **1_6_**, releasing the catalyst from the cage. As compound **10** can only be formed while the cationic gold species is encapsulated in **1_6_**, its production stopped, whereas formation of **11** started again. The formation of product **12** followed the same trend as **11**, but it was less clear as this compound was formed in much smaller amounts. As ketone **9** can be formed both inside and outside the cage (Table 1), the formation of this product could not be switched; the faster formation of this product after addition of both cage and Et_4_N^+^BF_4_^−^ (**7**) corresponds with the observations in Table 1.

**Figure 1:**
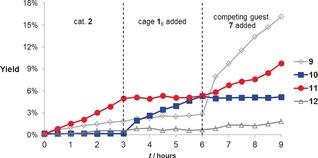
Formation of products from the reaction of substrate **8** in the presence of stepwise addition of cage and competing guest. Reaction conditions: [**8**]=66 mm, [**2**]=1.7 mm (2.5 mol %), [H_2_O]=44 mm, [**1**]=33 mm, [**7**]=33 mm. Yields are averaged from two measurements.

In conclusion, we have demonstrated that it is possible to control the pathway by which gold complexes convert substrate molecules by complex encapsulation events. In the presence of a self‐assembled hexameric resorcin[4]arene cage the dinuclear complex [{Au(NHC)}_2_(μ‐OH)][X] breaks into mononuclear units that are encapsulated as the dinuclear gold complex **2** is too large to fit inside the cage. By doing so, the dual activation reactivity typical for complex **2** is switched into (re)activity that is typical for mononuclear complexes. By using this strategy on a reaction that can only proceed through a dual‐activation pathway, we have demonstrated on/off switching of a gold‐catalyzed reaction. Moreover, this approach also provides tools to change the product distribution during the course of a reaction, which is demonstrated in the activation of 4‐phenyl‐1‐butyne. In summary, this strategy of switching the active species of a gold‐catalyzed reaction by means of host‐guest interactions provides a new approach to controlling catalytic transformations, even during the course of the reaction.

## Experimental Section

**Hydrophenoxylation experiments**: The catalyst **2** (0.025 mol %) was added to a solution of **4** (550 mm) and **5** (500 mm) and in selected experiments cage **1**_6_ (50 mm) and/or competing guest **7** (50 mm) in [D_8_]toluene. The mixture was heated to 80 °C and yields were monitored using GC and ^1^H NMR of the crude mixture.

**Catalytic experiments with substrate 8**: The catalyst **2** (2.5 mol %), H_2_O (44 mm), substrate **8** (66 mm), and, in selected experiments, cage **1**_6_ (33 mm) and/or competing guest **7** (33 mm) were mixed in [D_6_]benzene and heated to 70 °C for 48 h. Yields were monitored using GC and ^1^H NMR of the crude mixture and were determined as the average of two experiments.

## Supporting information

As a service to our authors and readers, this journal provides supporting information supplied by the authors. Such materials are peer reviewed and may be re‐organized for online delivery, but are not copy‐edited or typeset. Technical support issues arising from supporting information (other than missing files) should be addressed to the authors.

Supplementary

## References

[1] For selected reviews on catalysis in confined spaces, see:

[1a] M. Yoshizawa , J. K. Klosterman , M. Fujita , Angew. Chem. Int. Ed. 2009, 48, 3418–3438; 10.1002/anie.200805340 19391140

[1b] M. J. Wiester , P. A. Ulmann , C. A. Mirkin , Angew. Chem. Int. Ed. 2011, 50, 114–137; 10.1002/anie.201000380 20922725

[1c] M. Raynal , P. Ballester , A. Vidal-Ferran , P. W. N. M. van Leeuwen , Chem. Soc. Rev. 2014, 43, 1734–1787; 24365792 10.1039/c3cs60037h

[1d] S. Zarra , D. M. Wood , D. A. Roberts , J. R. Nitschke , Chem. Soc. Rev. 2015, 44, 419–432; 25029235 10.1039/c4cs00165f

[1e] S. H. A. M. Leenders , R. Gramage-Doria , B. de Bruin , J. N. H. Reek , Chem. Soc. Rev. 2015, 44, 433–448; 25340992 10.1039/c4cs00192c

[1f] C. J. Brown , F. D. Toste , R. G. Bergman , K. N. Raymond , Chem. Rev. 2015, 115, 3012–3035; 25898212 10.1021/cr4001226

[1g] O. Bistri , O. Reinaud , Org. Biomol. Chem. 2015, 13, 2849–2865. 25608497 10.1039/c4ob02511c

[2a] V. F. Slagt , J. N. H. Reek , P. C. J. Kamer , P. W. N. M. Van Leeuwen , Angew. Chem. Int. Ed. 2001, 40, 4271–4274; 10.1002/1521-3773(20011119)40:22<4271::AID-ANIE4271>3.0.CO;2-H 29712091

[2b] V. F. Slagt , P. C. J. Kamer , P. W. N. M. van Leeuwen , J. N. H. Reek , J. Am. Chem. Soc. 2004, 126, 1526–1536; 14759211 10.1021/ja0386795

[2c] M. Kuil , T. Soltner , P. W. N. M. van Leeuwen , J. N. H. Reek , J. Am. Chem. Soc. 2006, 128, 11344–11345; 16939244 10.1021/ja063294i

[2d] V. Bocokić , A. Kalkan , M. Lutz , A. L. Spek , D. T. Gryko , J. N. H. Reek , Nat. Commun. 2013, 4, 2670. 24150228 10.1038/ncomms3670

[3] D. Fiedler , D. H. Leung , R. G. Bergman , K. N. Raymond , Acc. Chem. Res. 2005, 38, 349–358. 15835881 10.1021/ar040152p

[4] D. H. Leung , R. G. Bergman , K. N. Raymond , J. Am. Chem. Soc. 2007, 129, 2746–2747. 17302420 10.1021/ja068688o

[5a] Z. J. Wang , C. J. Brown , R. G. Bergman , K. N. Raymond , F. D. Toste , J. Am. Chem. Soc. 2011, 133, 7358–7360; 21517023 10.1021/ja202055v

[5b] D. M. Kaphan , M. D. Levin , R. G. Bergman , K. N. Raymond , F. D. Toste , Science 2015, 350, 1235–1238. 26785485 10.1126/science.aad3087

[6] W. M. Hart-Cooper , K. N. Clary , F. D. Toste , R. G. Bergman , K. N. Raymond , J. Am. Chem. Soc. 2012, 134, 17873–17876. 23066637 10.1021/ja308254k

[7] D. G. H. Hetterscheid , S. H. Chikkali , B. de Bruin , J. N. H. Reek , ChemCatChem 2013, 5, 2785–2793.

[8] E. B. Walczuk , P. C. J. Kamer , P. W. N. M. van Leeuwen , Angew. Chem. Int. Ed. 2003, 42, 4665–4669; 10.1002/anie.200351884 14533158

[9] S. H. Hong , M. W. Day , R. H. Grubbs , J. Am. Chem. Soc. 2004, 126, 7414–7415. 15198568 10.1021/ja0488380

[10] M. Otte , P. F. Kuijpers , O. Troeppner , I. Ivanović-Burmazović , J. N. H. Reek , B. de Bruin , Chem. Eur. J. 2013, 19, 10170–10178. 23821458 10.1002/chem.201301411

[11] For overviews of switchable catalysis, see:

[11a] U. Lüning , Angew. Chem. Int. Ed. 2012, 51, 8163–8165; 10.1002/anie.201204567 22821724

[11b] T. Imahori , S. Kurihara , Chem. Lett. 2014, 43, 1524–1531;

[11c] V. Blanco , D. A. Leigh , V. Marcos , Chem. Soc. Rev. 2015, 44, 5341–5370; 25962337 10.1039/c5cs00096c

[11d] A. J. McConnell , C. S. Wood , P. P. Neelakandan , J. R. Nitschke , Chem. Rev. 2015, 115, 7729–7793. 25880789 10.1021/cr500632f

[12] G. Bianchini , A. Scarso , G. La Sorella , G. Strukul , Chem. Commun. 2012, 48, 12082–12084. 10.1039/c2cc37374b 23135428

[13] A. Cavarzan , A. Scarso , P. Sgarbossa , G. Strukul , J. N. H. Reek , J. Am. Chem. Soc. 2011, 133, 2848–2851. 21319822 10.1021/ja111106x

[14] L. R. Macgillivray , J. L. Atwood , Nature 1997, 389, 469–472.

[15] A. Shivanyuk , J. Rebek, Jr. , Proc. Natl. Acad. Sci. 2001, 98, 7662–7665. 11427733 10.1073/pnas.141226898 PMC35398

[16] D. J. Gorin , B. D. Sherry , F. D. Toste , Chem. Rev. 2008, 108, 3351–3378. 18652511 10.1021/cr068430g PMC2754803

[17] S. Handa , S. S. Subramanium , A. A. Ruch , J. M. Tanski , L. M. Slaughter , Org. Biomol. Chem. 2015, 13, 3936. 25711170 10.1039/c4ob02640c

[18] M. Galli , J. E. M. Lewis , S. M. Goldup , Angew. Chem. Int. Ed. 2015, 54, 13545—13549; 10.1002/anie.201505464 PMC4678423 26387887

[19a] R. S. Ramón , S. Gaillard , A. Poater , L. Cavallo , A. M. Z. Slawin , S. P. Nolan , Chem. Eur. J. 2011, 17, 1238–1246; 21243690 10.1002/chem.201002607

[19b] A. Gómez-Suárez , Y. Oonishi , S. Meiries , S. P. Nolan , Organometallics 2013, 32, 1106–1111.

[20] S. P. Nolan , Acc. Chem. Res. 2011, 44, 91–100. 21028871 10.1021/ar1000764

[21] Y. Oonishi , A. Gómez-Suárez , A. R. Martin , S. P. Nolan , Angew. Chem. Int. Ed. 2013, 52, 9767–9771; 10.1002/anie.201304182 23897673

[22] L. Adriaenssens , A. Escribano-Cuesta , A. Homs , A. M. Echavarren , P. Ballester , Eur. J. Org. Chem. 2013, 1494–1500.

[23] A. S. K. Hashmi , Gold Bull. 2003, 36, 3–9.

[24] G. C. Fortman , A. Poater , J. W. Levell , S. Gaillard , A. M. Z. Slawin , I. D. W. Samuel , L. Cavallo , S. P. Nolan , Dalton Trans. 2010, 39, 10382–10390. 20922211 10.1039/c0dt00276c

[25] P. H. Y. Cheong , P. Morganelli , M. R. Luzung , K. N. Houk , F. D. Toste , J. Am. Chem. Soc. 2008, 130, 4517–4526. 18327944 10.1021/ja711058f PMC2995695

[26a] I. Braun , A. M. Asiri , A. S. K. Hashmi , ACS Catal. 2013, 3, 1902–1907;

[26b] A. S. K. Hashmi , T. Lauterbach , P. Nösel , M. H. Vilhelmsen , M. Rudolph , F. Rominger , Chem. Eur. J. 2013, 19, 1058–1065; 23255064 10.1002/chem.201203010

[26c] A. S. K. Hashmi , Acc. Chem. Res. 2014, 47, 864–876. 24533563 10.1021/ar500015k

[27a] L. Ye , Y. Wang , D. H. Aue , L. Zhang , J. Am. Chem. Soc. 2012, 134, 31–34; 22176593 10.1021/ja2091992 PMC3257394

[27b] Y. Wang , A. Yepremyan , S. Ghorai , R. Todd , D. H. Aue , L. Zhang , Angew. Chem. Int. Ed. 2013, 52, 7795–7799; 10.1002/anie.201301057 PMC4120750 23788447

[28a] A. Gómez-Suárez , Y. Oonishi , A. R. Martin , S. V. C. Vummaleti , D. J. Nelson , D. B. Cordes , A. M. Z. Slawin , L. Cavallo , S. P. Nolan , A. Poater , Chem. Eur. J. 2016, 22, 1125–1132; 26662656 10.1002/chem.201503097

[28b] A. Gómez-Suárez , Y. Oonishi , A. R. Martin , S. P. Nolan , Beilstein J. Org. Chem. 2016, 12, 172–178. 26977176 10.3762/bjoc.12.19 PMC4778530

[29] The fact that the reaction between diphenylacetylene and phenol does not take place in the presence of the cage could also be caused by the unability of the substrates and catalyst to coencapsulate inside the cage. However, the reaction between diphenylacetylene and thiophenol, which can be catalyzed by a mononuclear, π-activating catalyst due to the stronger nucleophile, did yield the coupling product. This shows that coencapsulation of the two substrates and the catalyst is possible and the hydrophenoxylation is switched off due to preventing the dinuclear pathway. For references on hydrothiolation:

[29a] X. Zhang , K. Wang , RSC Adv. 2015, 5, 34439–34446;

[29b] T. Tamai , K. Fujiwara , S. Higashimae , A. Nomoto , A. Ogawa , Org. Lett. 2016, 18, 2114–2117. 27057590 10.1021/acs.orglett.6b00746

[30] S. Sun , J. Kroll , Y. Luo , L. Zhang , Synlett 2012, 54–56. 22904604 10.1055/s-0031-1289567 PMC3419542

[31] N. Marion , R. S. Ramón , S. P. Nolan , J. Am. Chem. Soc. 2009, 131, 448–449. 19140786 10.1021/ja809403e

[32] A. Leyva , A. Corma , J. Org. Chem. 2009, 74, 2067–2074. 19170603 10.1021/jo802558e

