# Supplementary material for: A Switchable Gold Catalyst by Encapsulation in a Self‐Assembled Cage
Source: Chemistry. 2016 Sep 4;22(42):14836–9. doi: 10.1002/chem.201603162 (PMC5053284; doi:10.1002/chem.201603162)
Supplement: Supplementary file 1 — Supplementary [file CHEM-22-14836-s001.pdf]

CHEMISTRY
A European Journal

Supporting Information

# A Switchable Gold Catalyst by Encapsulation in a Self-Assembled Cage

Anne C. H. Jans,[a] Adrián Gómez-Suárez,[b] Steven P. Nolan,[c, d] and Joost N. H. Reek*[a]

chem_201603162_sm_miscellaneous_information.pdf

### General

$^1H$ NMR spectra were measured on a Bruker AMX 400 and spectral data were referenced to residual protiated solvents (7.26 ppm for $CDCl_3$). 2D $^1H$ DOSY NMR experiments were performed on a DRX 300 with temperature and gradient calibration prior to the measurements, at a constant temperature of 298 K. All reagents were purchased from commercial suppliers and used without further purification. [{Au(IPr)}$_2$(μ-OH)][$BF_4$][1] and resorcin[4]arene[2] were prepared according to literature procedures. All the products formed during the catalytic reactions were identified by $^1H$ NMR and GC analysis.

### NMR experiments

Encapsulation of [{Au(IPr)}$_2$(μ-OH)][$BF_4$] in resorcin[4]arene hexamer

In a typical encapsulation experiment, [{Au(IPr)}$_2$(μ-OH)][$BF_4$] (3.58 mg, $2.81 \cdot 10^{-3}$ mmol, 0.5 eq.) was added to a solution of the resorcin[4]arene (37.3 mg, $33.7 \cdot 10^{-3}$ mmol, 6.0 eq.) in $H_2O$ saturated $CDCl_3$ (0.6 mL). The mixture was stirred for 15 minutes at RT. Encapsulation was observed by $^1H$ NMR (Figure S1) and 2D DOSY $^1H$ NMR (Figure S2).

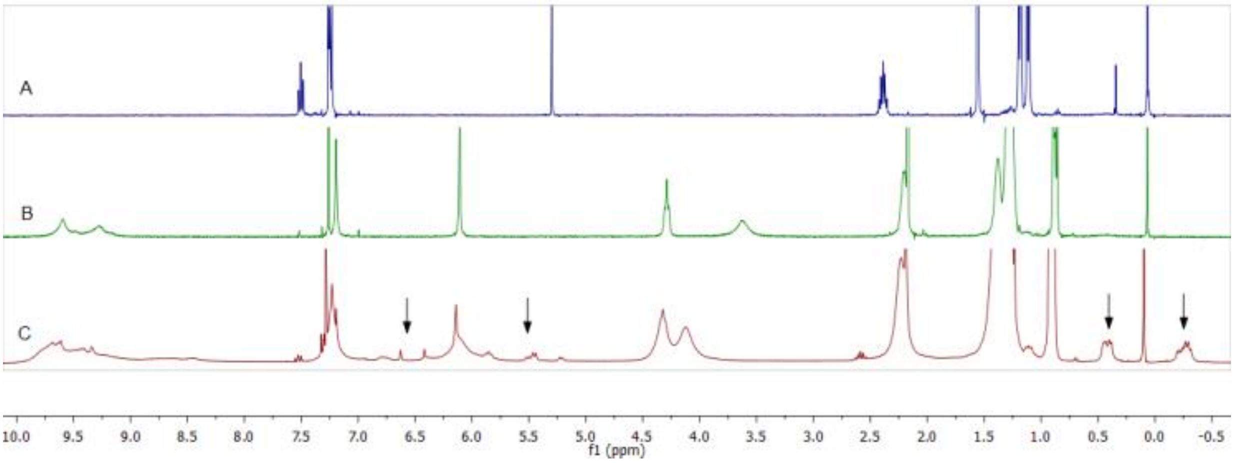


**Figure S1.** $^1H$ NMR spectra in $H_2O$ saturated $CDCl_3$. A) Dinuclear gold complex **2**. B) Cage $\mathbf{1}_6$. C) Combination of cage $\mathbf{1}_6$ and complex **2**. Arrows indicate proton signals of the encapsulated species.

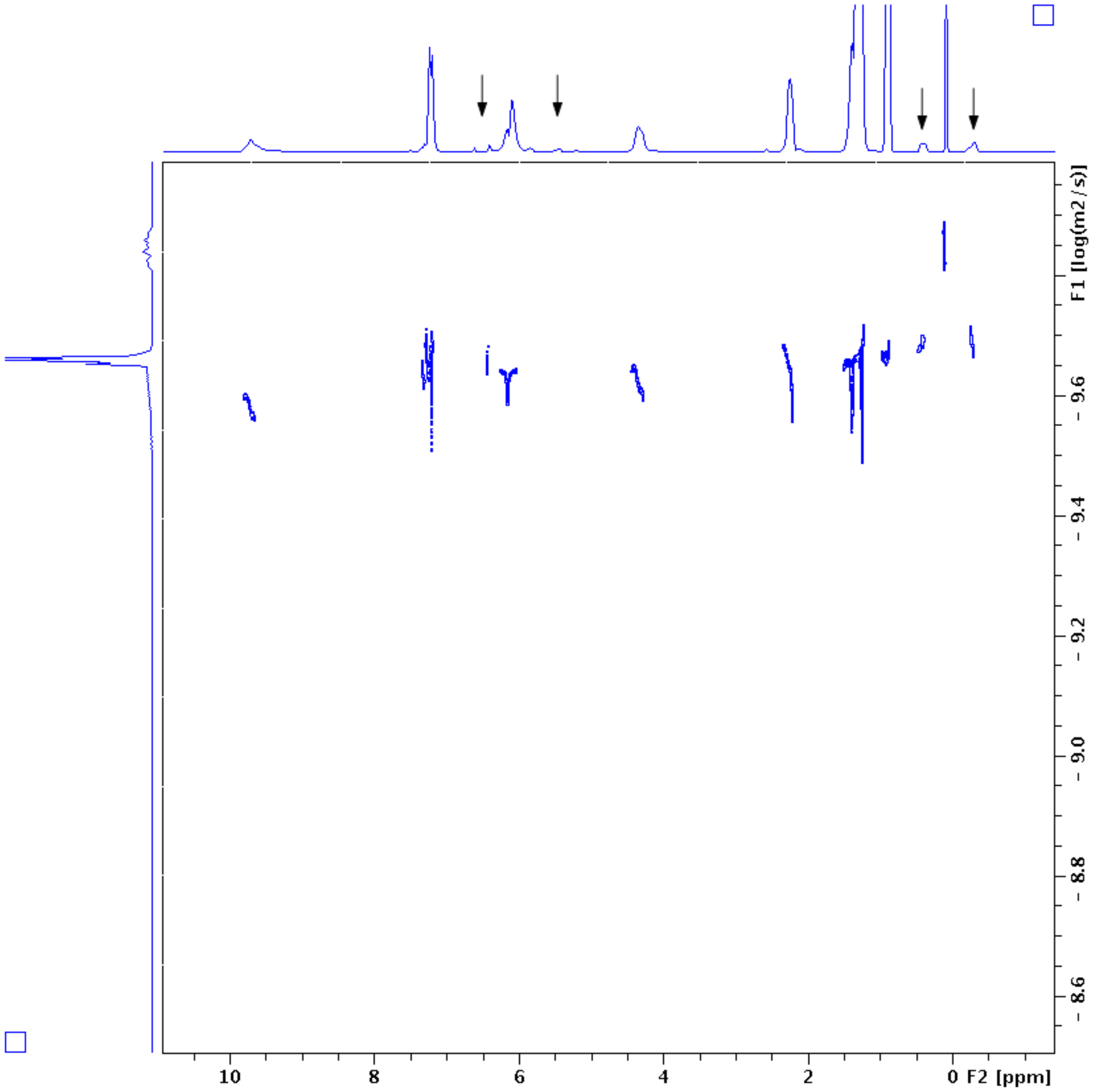


**Figure S2.** $^{1}$H 2D DOSY NMR plot of encapsulated **2** in $H_2O$ saturated $CDCl_3$ (**1**$_6$ : **2** = 2 : 1). All signals have the same diffusion constant, indicating encapsulation of the complex. Arrows indicate proton signals of the encapsulated species.

An encapsulation experiment was also performed in deuterated benzene (Figure S3), but usage of this solvent leads to less well resolved NMR spectra. However, encapsulation of the complex was still obtained.

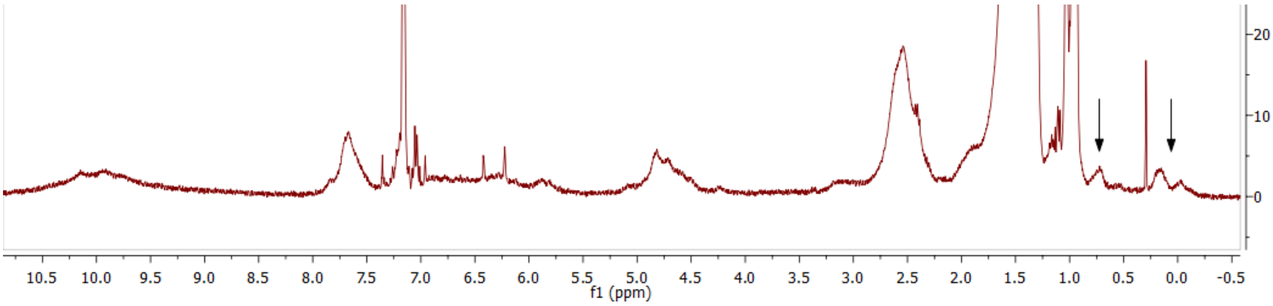


**Figure S3.** $^{1}$H NMR spectrum of the combination of cage **1**$_6$ and complex **2** in benzene-d6. Arrows indicate proton signals of the encapsulated species.

**Catalytic experiments**

Hydrophenoxylation reaction in absence of cage

The reaction was performed in a 3 mL vial under air. [{Au(IPr)}$_2$(μ-OH)][$BF_4$] (2.39 mg, $1.88 \cdot 10^{-3}$ mmol, $0.25 \cdot 10^{-3}$ eq.) was added to a solution of diphenylacetylene (66.8 mg, 0.375 mmol, 1.0 eq.) and phenol (36.2 μL, 38.8 mg, 0.4125 mmol, 1.1 eq.) in toluene-d8 (0.75 mL). The mixture was heated to 80°C and stirred for 60 minutes. Conversion was monitored using GC and $^1$H NMR of the crude mixture. Characterization data of the formed products match those reported in the literature.[3]

Hydrophenoxylation reaction in presence cage

The reaction was performed in a 3 mL vial under air. The resorcin[4]arene (41.6 mg, $37.6 \cdot 10^{-3}$ mmol, $5.0 \cdot 10^{-3}$ eq.) was dissolved in toluene-d8 (0.75 mL) by shortly heating the mixture under hot tap water. [{Au(IPr)}$_2$(μ-OH)][$BF_4$] (2.39 mg, $1.88 \cdot 10^{-3}$ mmol, $0.25 \cdot 10^{-3}$ eq.) was added and the mixture was stirred for 20 minutes at RT to ensure full encapsulation of the complex. Then, diphenylacetylene (66.8 mg, 0.375 mmol, 1.0 eq.) and phenol (36.2 μL, 38.8 mg, 0.4125 mmol, 1.1 eq.) were added. Mesitylene (52.2 μL, 45.1 mg, 0.375 mmol, 1.0 eq.) was added as an internal standard. The mixture was heated to 80°C and stirred for 24 hours. Conversion was monitored using GC and $^1$H NMR of the crude mixture. After 24 hours, $Et_4N^+BF_4^-$ (8.16 mg, $37.6 \cdot 10^{-3}$ mmol, $5.0 \cdot 10^{-3}$ eq.) was added as a competing guest. The conversion was further monitored using GC and $^1$H NMR of the crude mixture.

Conversion of 4-phenyl-1-butyne in absence cage

The reaction was performed in a 3 mL vial under air. [{Au(IPr)}$_2$(μ-OH)][$BF_4$] (1.58 mg, $1.24 \cdot 10^{-3}$ mmol, 0.025 eq.), $H_2O$ (0.59 μL, $32.7 \cdot 10^{-3}$ mmol, 0.67 eq.), 4-phenyl-1-butyne (6.96 μL, 6.44 mg, $49.5 \cdot 10^{-3}$ mmol, 1.0 eq.) and mesitylene (6.89 μL, 5.95 mg, $49.5 \cdot 10^{-3}$ mmol, 1.0 eq.) were added to $C_6D_6$ (0.75 mL). The mixture was heated to 70°C and stirred for 48 hours. Conversion was monitored using GC and $^1$H NMR of the crude mixture and was determined as the average of two experiments. Characterization data matched those found in literature.[4]

Conversion of 4-phenyl-1-butyne in presence cage

The reaction was performed in a 3 mL vial under air. The resorcin[4]arene (27.4 mg, $24.8 \cdot 10^{-3}$ mmol, 0.50 eq.) was dissolved in $C_6D_6$ (0.75 mL) and $H_2O$ (0.59 μL, $32.7 \cdot 10^{-3}$ mmol, 0.67 eq.) by shortly heating the mixture under hot tap water. [{Au(IPr)}$_2$(μ-OH)][$BF_4$] (1.58 mg, $1.24 \cdot 10^{-3}$ mmol, 0.025 eq.) was added and the mixture was stirred for 20 minutes at RT to ensure full encapsulation of the complex. Then, 4-phenyl-1-butyne (6.96 μL, 6.44 mg, $49.5 \cdot 10^{-3}$ mmol, 1.0 eq.) was added. Mesitylene (6.89 μL, 5.95 mg, $49.5 \cdot 10^{-3}$ mmol, 1.0 eq.) was added as an internal standard. The mixture was heated to 70°C and stirred for 48 hours. Conversion was monitored using GC and $^1$H NMR of the crude mixture and was determined as the average of two experiments.

Switching experiment

The reaction was performed in a 3 mL vial under air. [{Au(IPr)}$_2$(μ-OH)][$BF_4$] (3.16 mg, $2.48 \cdot 10^{-3}$ mmol, 0.025 eq.), $H_2O$ (1.19 μL, $65.8 \cdot 10^{-3}$ mmol, 0.67 eq.), 4-phenyl-1-butyne (13.92 μL, 12.89 mg, $99.0 \cdot 10^{-3}$ mmol, 1.0 eq.) and mesitylene (13.77 μL, 11.90 mg, $99.0 \cdot 10^{-3}$ mmol, 1.0 eq.) were added to $C_6D_6$ (1.5 mL). The mixture was heated to 70°C and stirred for 3 hours. A sample for GC was taken every 30 minutes from the crude mixture.

After 3 hours, the resorcin[4]arene (54.73 mg, $49.5 \cdot 10^{-3}$ mmol, 0.50 eq.) was added to the mixture, and the progress of the reaction was monitored by making samples for GC-analysis every 30 minutes. Conversion was determined as the average of two experiments. After the next 3 hours (6 h after initiating the experiment), $Et_4N^+BF_4^-$ (10.7 mg, $49.5 \cdot 10^{-3}$ mmol, 0.050 eq.) was added as a competing guest. The reaction was continued and samples for GC were taken every 30 minutes. This experiment was repeated and the data displayed are average conversions of two separate reactions (Figure 1). The faster formation of product **9** after addition of both cage and $Et_4N^+BF_4^-$ (**7**) is in correspondence with the observations displayed in Table 1.

**Figure S4.** Formation of products in the gold-catalyzed conversion of 4-phenyl-1-butyne (**7**) in presence of stepwise addition of cage and competing guest.

Hydrothiolation of diphenylacetylene in absence of cage

The reaction was performed in a 3 mL vial under air. Diphenylacetylene (53.6 mg, 0.301 mmol, 1.0 eq.) and thiophenol (33.9 µL, 36.4 mg, 0.330 mmol, 1.1 eq.) were added to a mixture of IPr-Au-$NTf_2$ (2.63 mg, $3.03 \cdot 10^{-3}$ mmol, 0.010 eq.) and $H_2O$ (0.70 µL, 0.039 mmol, 0.13 eq.) in toluene-d8 (0.60 mL). Mesitylene (41.7 µL, 36.0 mg, 0.300 mmol, 1.0 eq.) was added as an internal standard. The mixture was stirred at 80°C for 60 minutes and the products were analyzed using $^1$H NMR, GC and MS. Characterization data of the formed product match those reported in the literature.[5]

Hydrothiolation of diphenylacetylene in presence of cage

The reaction was performed in a 3 mL vial under air. The resorcin[4]arene (33.2 mg, 0.0300 mmol, 0.010 eq.) was dissolved in toluene-d8 (0.60 mL) and $H_2O$ (0.70 µL, 0.039 mmol, 0.13 eq.) by shortly heating the mixture under hot tap water. IPr-Au-$NTf_2$ (2.64 mg, $3.04 \cdot 10^{-3}$ mmol, 0.010 eq.) was added and the mixture was stirred for 20 minutes at RT to ensure full encapsulation of the complex. Then, diphenylacetylene (53.6 mg, 0.301 mmol, 1.0 eq.) and thiophenol (33.9 µL, 36.4 mg, 0.330 mmol, 1.1 eq.) were added. Mesitylene (41.7 µL, 36.0 mg, 0.300 mmol, 1.0 eq.) was added as an internal standard. The mixture was stirred at 80°C for 60 minutes and the products were analyzed using $^1$H NMR, GC and MS.

**References**


[1] A. Gómez-Suárez, Y. Oonishi, S. Meiries, S. P. Nolan, *Organometallics* **2013**, *32*, 1106–1111.

[2] I. Elidrisi, S. Negin, P. V Bhatt, T. Govender, H. G. Kruger, G. W. Gokel, G. E. M. Maguire, *Org. Biomol. Chem.* **2011**, *9*, 4498–4506.

[3] Y. Oonishi, A. Gómez-Suárez, A. R. Martin, S. P. Nolan, *Angew. Chem. Int. Ed.* **2013**, *52*, 9767–9771.

[4] S. Sun, J. Kroll, Y. Luo, L. Zhang, *Synlett* **2012**, *23*, 54–56.

[5] I. G. Trostyanskaya, I. P. Beletskaya, *Synlett* **2012**, 535–540.
